# Underwater Impact and Intention–Behaviour Gap of Scuba Divers on Coral Communities in Hong Kong SAR, China

**DOI:** 10.3390/ijerph20053896

**Published:** 2023-02-22

**Authors:** Jun-Yin So, Ying Kwok, Christie Lai, Hei-Wut Fong, Lee-Yan Pang

**Affiliations:** 1 WWF-Hong Kong, Hong Kong SAR, China; 2 School of Biological Sciences, The University of Hong Kong, Pok Fu Lam, Hong Kong SAR, China; 3 School of Life Sciences, The Chinese University of Hong Kong, Shatin, Hong Kong SAR, China

**Keywords:** scuba diving, environmental impact, citizen science, corals, marine biota

## Abstract

Recreational diving, under the continual growth of the scuba diving industry, may escalate coral reef damage as one of the substantial anthropogenic impacts and is of pressing concern. Besides unregulated and excessive diving activities, accidental contact with corals by inexperienced divers can cause recurring physical damage and heighten the pressure on coral communities. Understanding the ecological impacts of underwater contact with marine biota will thus be crucial to develop more sustainable scuba diving practices in Hong Kong. To probe the scuba diving impacts of divers’ contact with coral communities, WWF-Hong Kong started a citizen science monitoring programme and invited 52 advanced divers to conduct direct underwater observations. Questionnaires were also developed to examine and address the research gap between the associated attitudes and the perceived contact rate of divers. Results from analysing the underwater behaviours of 102 recreational divers showed inconsistent perceived and actual contact rates. It was revealed that recreational divers might often overlook the ecological effects of their activities underwater on coral communities. The questionnaire findings will be utilised to improve the framework of the dive-training programmes and enhance divers’ awareness to minimise their influence on the marine environment.

## 1. Introduction

Scuba diving is one of the most popular nature-based water sports in the world [1], with more than 1 million new divers being certified by the Professional Association of Diving Instructors (PADI) in 2018 [2]. The significant expansion of the scuba diving industry can be ascribed to several factors: improved diving safety, growing interest in nature-based recreational activities, and more convenience in visiting overseas dive destinations [3]. With the continuous growth of the scuba diving industry, concerns about its ecological impact have been raised [4,5,6].

Direct and indirect damage inflicted by intensive recreational diving will affect fragile coral reefs. Studies of diver impact in the Red Sea, Australia, the Caribbean, and Mexico [4,7,8,9,10,11] have indicated that exhaustive and unrestricted diving activities induce negative effects on marine organisms, through direct contact by divers (i.e., body parts or dive equipment such as fins and camera) [7,12,13] and anchoring [14]. Such contact causes breakage of the skeletal structure or the loss of soft tissue of benthic organisms, especially corals, sponges, and bryozoans. Apart from direct contact, these activities increase the resuspension of benthic sediment, posing indirect health impacts on benthic organisms [15,16]. If unregulated, the cumulative impact would adversely alter the community structure of benthic organisms in coral reefs [17,18]. With climate change inducing more frequent extreme weather events, compounded by additional anthropogenic pressure from the rapid growth of the diving industry, the future health of the marine ecosystem and its adaptability to new environmental changes is worrying. With these considerations, an assessment of the divers’ profiles and their impacts on the coral community in Hong Kong, with recommendations on an enhanced code of conduct in scuba diving is warranted.

Hong Kong SAR, China is located in the subtropical region of the South China Sea (22°8′–22°35′ N, 113°49′–114°31′ E), with seasonal fluctuations of water temperature ranging from 29 °C in summers down to 14 °C in winters, which presents a marginal and stressful environment for coral growth [19,20,21]. Despite not having the optimal environment for forming continuous reef structures, at least 84 scleractinian coral species have been recorded, forming non-reefal coral communities in Hong Kong [22,23]. The major coral areas are distributed in the eastern and northeastern coastal regions of Hong Kong, comprising 59 ± 12.6% and 33.33 ± 13.8% of coral coverage, respectively [24]. Coral cover varied substantially, ranging from 2.1% to 79.1% across 41 study sites in Hong Kong [24]. Hong Kong’s corals are not forming the reefal environment, yet it sustains high marine biodiversity. Indeed, Hong Kong was continuously discovering new coral species [25] and coral-associated species such as fishes [26], nudibranchs [27,28], and crabs [29].

With the outbreak of COVID-19 in 2019, international travel came to a halt and Hong Kong residents were restricted to stay and spend locally, subsequently increasing the need for local nature-based tourism significantly [30]. This led to the bloom of marine-based tourism, giving rise to the popularity of dive training courses and same-day scuba diving trips, and coral areas attracted unprecedented high usage from recreational diving and snorkelling activities [31,32]. Such intense underwater activity is of grave concern as beginners and inexperienced divers may accidentally contact corals, causing continuing physical damage to the benthic environment. Hong Kong currently does not have clear guidelines and regulations for scuba diving training courses and recreational diving activities with the aim to protect habitats and marine life.

Any ecosystem that has reached beyond its carrying capacity is unsustainable; empirical evidence has also shown that the ecological carrying capacities of the ocean in China [33], along with many renowned dive sites in Palau [34], Indonesia [35], Malaysia [36], and the Persian Gulf [37] are already overloaded. Ecological carrying capacity is an indicator that is widely used in the environmental management of recreational activities. Although there is no universally accepted definition of carrying capacity due to highly conditional-based measuring parameters [38], it can be generally defined as “the ability of a resource to resist recreational use without unacceptable damage to its ecological components” [39]. In Hong Kong, most of the diving activities are concentrated in 33 dive sites [24,32]. It is notable that the carrying capacities of these dive sites have already been exceeded [40], given the limited diving area at each site and the growing number of divers.

Despite its popularity, the marine carrying capacity in Hong Kong is still largely understudied [40]. A four-dimensional approach study on carrying capacity measurements found that the most influential factors affecting marine carrying capacities are social and ecological [33]. It has been demonstrated that the sustainability of diving activities is dependent on both the number of divers at the site and the capacity of the ecosystem. If the threshold of carrying capacity is reached, the regenerative ability and recovery rate of these ecosystems, especially from bleaching events, will be weakened [34,41,42,43]. Few dive sites globally have established an ecological carrying capacity due to a lack of quantified behavioural data [36].

The availability of quantified behavioural data can provide strong evidence to determine the carrying capacity under the influence of tourism and identify how it can be improved. An essential finding in Hong Kong exhibited a statistically significant positive correlation between the number of divers visiting the site and the number of broken coral colonies [13]. Physical contact was the leading cause of biological impacts, with hands and fins being the most recurrent contact methods [4,7,8,9,10,11]. The way of contact could either be intentional or unintentional. Data showed that contact was mostly intentionally by hand and unintentionally by fins [44]. For many years, the lack of conservation awareness has been thought to be the culprit of this phenomenon, under the theory that behaviours are primarily driven by attitude [45,46]. WWF-Hong Kong conducted an online questionnaire survey in 2021 on 581 Hong Kong divers, and more than 90% of respondents agreed that divers are responsible for protecting the dive sites’ environments. Surveyed divers have also expressed considerations of their effects on dive sites during scuba diving. The results demonstrated an overall positive attitude and awareness toward environmental protection among Hong Kong divers. However, it raises the question of misalignment between behaviour and attitude. While it might be valid that under the theory of planned behaviour, intention leads to behaviour [47].

Perceived behavioural control could indeed affect both intention and behaviours [47]. Even so, different experiments discovered that one’s good intentions might not translate to one’s good behaviour, even if the subject has perceived oneself as doing so [48,49,50]. Many studies emphasise the factors affecting perceived behaviours, but there is a lack of study on the intention-behaviour gap. The study of So et al. (2021) was the first to uncover the intention–behaviour gap by assessing the perceived and actual clam harvesting behaviours in Hong Kong [49]. Actual behavioural control acts on an important role in environmental education which gives rise to the intention–behaviour gap [51,52,53]. Environmental education not only aims to raise public awareness, but also aims to transfer to actual behaviour change, but behaviour is difficult to measure [49,54,55,56].

This study thus sheds light on the intention–behaviour gap of scuba divers and hypothesises that there is no direct relationship between intention and actual behaviour [49]. We sought to collect the updated actual underwater behaviours of scuba divers in Hong Kong and understand the intention–behaviour gap within the scuba diving community. The result of this study has important implications for the formulation of on-site measures to minimise the diving impacts on local coral communities.

## 2. Materials and Methods

### 2.1. Data Collection

To enable the collection of a larger data set and serve as a form of public education to facilitate actual behaviour changes, this study applied a citizen science approach in data collection. A total of 52 experienced divers were selected to conduct the survey as citizen scientists, and all were required to attend a series of theoretical and practical training prior to the survey. A local coral expert was invited to deliver coral ecology and identification training. As the citizen scientists were the key to effectively assuring the quality and consistency of the collected data, two underwater survey skill training sessions were held to standardize their data collection technique and survey methodology.

The survey was conducted between June and December 2021. The diving behaviours of 102 anonymous recreational scuba divers were observed and recorded. Citizen scientists could conduct observational surveys on any commercial dive boats to minimise sampling bias, but to facilitate a smoother operation, WWF-Hong Kong sought the prior agreement of 6 dive operators to allow our survey to be conducted with their divers if the citizen scientists did not want to approach other dive operators by themselves. The onboard instructor or divemaster from cooperating dive operators notified recreational divers of the survey during pre-dive briefings, and divers were free to decide whether to partake in the study. The citizen scientists would again seek approval from the recruited diver before beginning the survey, thus all recruited divers were well informed that their diving behaviours were being observed and recorded during the entire dive and all the contact with marine biota or substrate was recorded on a WWF’s custom-made underwater slate [12]. A follow-up questionnaire survey was then completed by participating divers after the underwater survey to investigate the intention–behaviour gap.

### 2.2. Direct Observation Underwater Survey

A direct observation underwater survey was conducted to record the divers’ behaviours and the related contact with the marine biota and substrate. A pair of citizen scientists conducted a survey with an observed recreational diver, with one citizen scientist focused on recording the diver’s underwater behaviour data, while the other assisted and took photographs of coral damage if contact with corals were made. In comparison to the previous study by Chung et al. 2013 [12], our study also investigated whether the divers could intentionally control their behaviour when they were being observed.

To identify the relationship between the contact rate with marine biota and substrate and the dive stage with the highest contact rates, the survey divided a recreational dive into three stages, namely the descent stage, the roaming stage between descent and ascent stages, and the ascent stage [12,57]. The descent stage covered the period from the water surface to the bottom and the first five minutes of the dive [12]. The citizen scientists would descend before the recruited diver so that they could observe the whole descent stage and record any contact with the marine biota. During the roaming stage, citizen scientists stayed diagonally behind the targeted diver to observe any contact made with marine biota. If contact with coral communities and coral damage were observed, another citizen scientist would record the significant contact and take coral damage photos for the record. The ascent stage covered the period that the recruited diver decided to end the dive.

All contact with marine biota and substrate by any part of the recruited diver’s body and/or dive equipment was recorded as either “Intentional” or “Unintentional” [12]. Intentional contact was defined as direct contact that the diver reached out and looked at the object before touching it, including putting hands on the coral for stabilisation. Unaware contact was categorised as unintentional contact. Defining intentions could be subjective and could be interpreted differently by the citizen scientists. Therefore, all marginal or unclear contact was standardised as “Unintentional”. If the recruited diver made contact with any marine biota for a prolonged period, such as the gauge or second octopus dragging while finning too close to the sea floor, it would be counted as one contact to reduce data duplication. The substrate type of each contact, such as coral, rocks, sand, and mud was also recorded. Citizen scientists would also identify the affected coral species or the growth form if it was not able to identify and record whether the contact had caused any damage. If there was coral damage, the damaged size (<5 cm and 5–15 cm and >15 cm) was recorded and photographs were taken.

### 2.3. Perceived Questionnaire

A follow-up questionnaire survey was conducted after the underwater survey to collect factors that could affect contact incidents with marine biota [Appendix A]. It was divided into three parts: the first part comprised 13 questions related to demographic information, socio-economic background, diving profile, and diving experience. The second consisted of 4 statements related to the awareness of their own or others’ behaviours affecting the marine ecosystems, which required the diver to rate their agreement on a five-point Likert scale (−2 = ‘Strongly disagree’; +2 = ‘Strongly agree’). The final part was related to the perceived diving behaviours in the observed dive and to investigate whether the diver realised contact was made with the marine substrate or even caused coral damage. To investigate the difference between perceived and actual behaviour, the questions included the self-reported number of contact with marine biota, reasons for contact and the body part/equipment in contact with the marine biota.

### 2.4. Study Site

**Figure 1:**
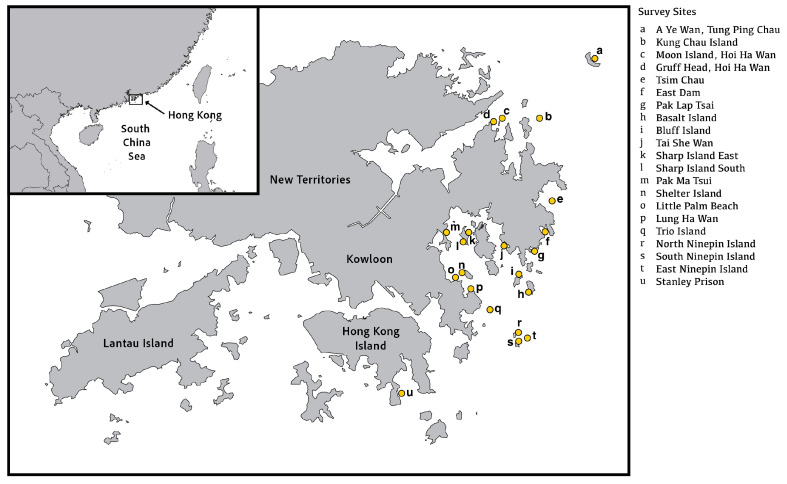
Map of Hong Kong SAR, China showing the survey sites.

The survey covered 21 local dive sites in the eastern part of Hong Kong, including A Ye Wan of Tung Ping Chau, Kung Chau Island, Moon Island and Gruff Head of Hoi Ha Wan, Tsim Chau, East Dam, Pak Lap Tsai, Basalt Island, Bluff Island, Tai She Wan, Sharp Island East and South, Pak Ma Tsui, Shelter Island, Little Palm Beach, Lung Ha Wan, Trio Island, Ninepin Island (North, East, South) and Stanley Prison (Figure 1).

### 2.5. Statistical Analysis

The software EXCEL was used to tabulate the data, and to detect statistical significance, analyses such as the normality test, Pearson Chi-Square, Ordinal regression, and Mann-Whitney U test were conducted as appropriate. SPSS v.23. was used to analyse and compute the significant level of employed statistical analyses.

## 3. Results

### 3.1. Sample Demographic

Demographic details of the sampled divers were in Table 1. Of the 102 divers observed by the citizen scientists, 48% (49 individuals) were female, 57% were aged between 20 and 34 years old, 34% were between 35 and 49 years old, and the remaining 9% were older than 50 years old. Divers with an advanced open water diver level qualification were the most frequently recruited (45%), followed by rescue diver level (18%) and open water diver (17%); 14% of the recruited divers had a level of instructor or above and the remaining 6% were at divemaster level.

### 3.2. Perceived and Actual Contact with Marine Biota

**Figure 2:**
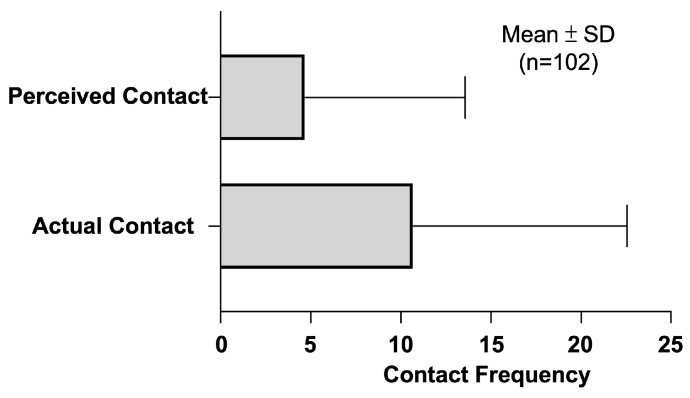
Bar plots for the mean of perceived and actual contact were shown above. The error bar represented one standard deviation. No significant difference was detected through the Mann–Whitney U test.

A total of 477 perceived contacts with marine biota were claimed by the recruited divers. The perceived contact for each diver ranged from 0 to 80 times. As for the actual contact, citizen scientists recorded over twice as many total actual contacts, reaching 1088 contacts. Actual contact for each diver ranged from 0 to 74 times. The means of perceived and actual contact were 4.68 ± 8.89 and 10.68 ± 11.89, respectively (Figure 2). No significant difference was detected through the Mann–Whitney U test.

**Figure 3:**
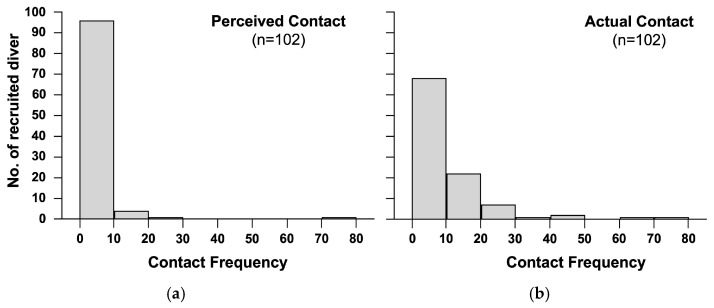
The histograms of the contact with marine biota and substrates for two types of contact: (**a**) perceived contact and (**b**) actual contact.

Histograms for both perceived contact and actual contact were plotted (Figure 3). Almost all divers perceived their number of contact as between 0 and 10. For the actual contact, the histogram indicated that the peak frequency still lay at the bin of 0 to 10. However, about a 30% reduction was observed for the peak frequency with more frequency distributed to the bin of 11 to 20 and others. The difference in the histograms implied the tendency to underestimate the marine substrate contact by divers. The Chi-Square test further supported the tendency of underestimation (Pearson Chi-Square = 25.742, *p* < 0.05).

### 3.3. Types of Contact and Contacted Body Parts/Equipment

**Figure 4:**
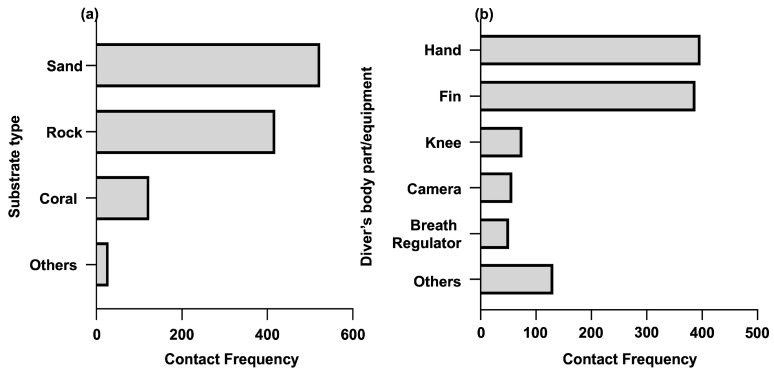
Contact frequency for substrate types and diver’s body parts: (**a**) bar plot for the contact frequency of substrates types is shown on the left. The *x*-axis shows the overall frequency for all the substrate types. The category of Others includes mud and silt. (**b**) bar plot for the contact frequency of the diver’s body parts is shown on the right. The *x*-axis shows the overall frequency for all the body parts. The category of Others includes the gauge and diving mud stick, etc.

Among the contact frequency of various substrates, sand and rock were the major types contacted by divers, where the frequencies were 523 (48%) and 418 (38%) respectively (Figure 4). Contact for corals was significantly lower at 124 times and no coral breakage was observed throughout the study. Regarding the body parts, the hand and fin were the dominant types. The frequency ranged from 388 (36%) to 397 (36%). Each of the remaining body parts accounted for about 5% of the frequency, except Others with about 10% of the total frequency.

**Figure 5:**
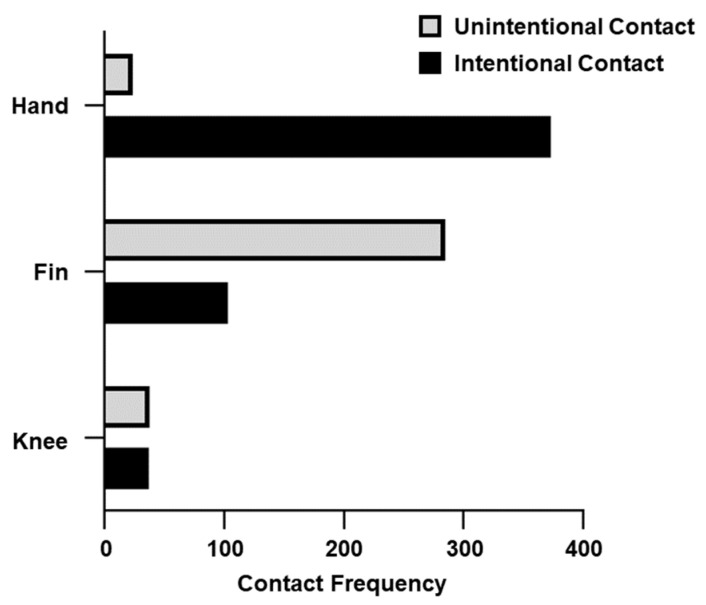
Unintentional contact and intentional contact for the top three contacted body parts (i.e., hand, fin, and knee). Unintentional contact is presented as the light grey bar, whereas intentional contact is in black.

Three frequently contacted body parts (i.e., hand, fin, and knee) were extracted and visualized in Figure 5. The contact was divided into “Unintentional” and “Intentional”. Among the hand contact, the majority was recorded as intentional (94%). However, both the fin and knee had a higher proportion of unintentional contact, which ranged from 51% to 73%.

### 3.4. Contact at Three Dive Stages

**Figure 6:**
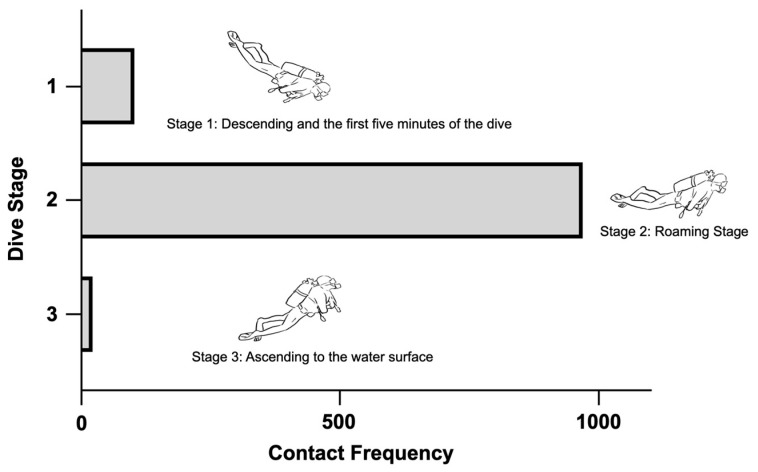
Contact frequency among three dive stages. The descent stage included the process of descending and the first five minutes of the dive. The roaming stage was the diving process after the descent stage. The ascent stage included the contact during the ascent to the water’s surface.

The contact frequencies were divided into three stages, the descent, roaming, and ascent stages (Figure 6). Among the three dive stages, 969 contacts (89%) were found in the roaming stage, when divers had reached the bottom and started their diving journey. Contact at both the descent and ascent stages accounted for 11% of the total contact.

**Figure 7:**
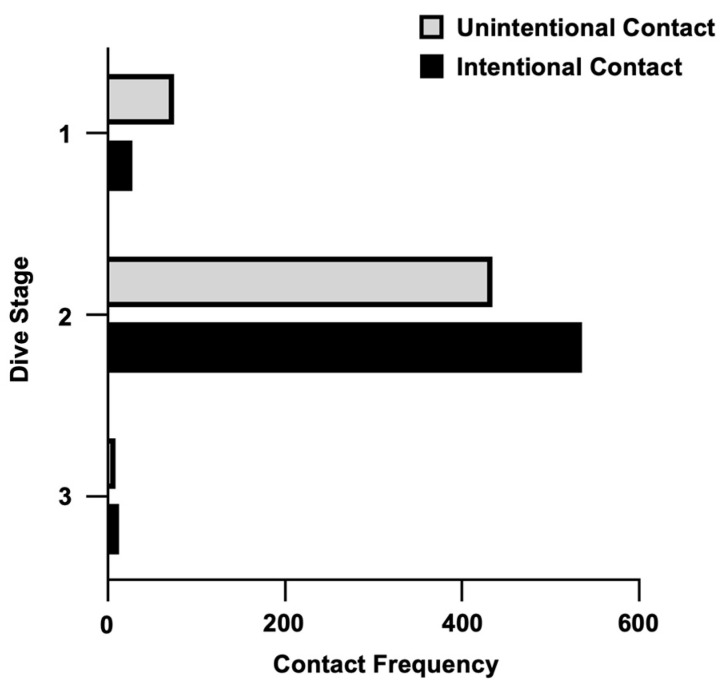
Unintentional and intentional contact frequency among three dive stages.

To further understand the relationship between the dive stage and the contact, the contact frequencies were further divided into “Unintentional” and “Intentional”. The roaming stage was found to have more intentional contact, the difference was notable, and the intentional contact was about 18% more than unintentional contact (Figure 7). In comparison to the ascent stage, the descent stage was found to have more unintentional contact, which was 70% more.

### 3.5. Coral Protection against Dive Certification

**Figure 8:**
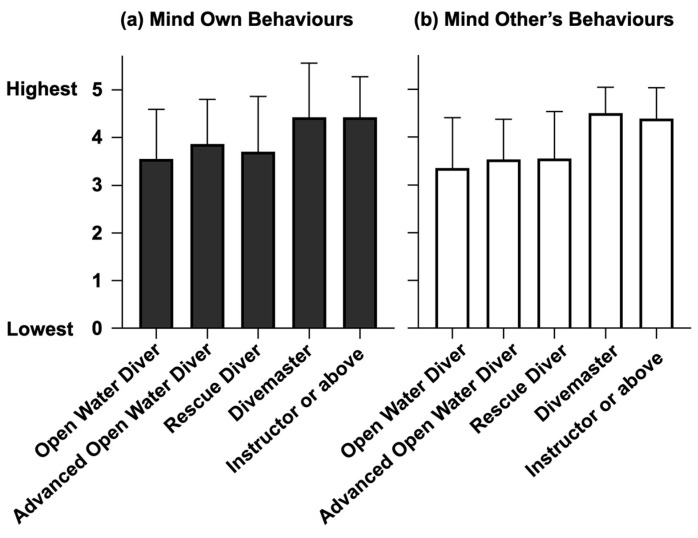
Awareness score to mind own behaviours and others’ behaviours. (**a**) the awareness score to mind own behaviour is shown on the left. The bar plots are separated by the divers’ certification from “0” (the lowest awareness) to “5” (the highest awareness). (**b**) the awareness score to mind others’ behaviours is shown on the right. The bar plots are separated by the dive certification from 0 (the lowest awareness) to 5 (the highest awareness).

The awareness of diver’s behaviours was divided into two groups: the awareness of own behaviours and the awareness of other’s behaviours (Figure 8). Divers with the qualification of divemaster or above generally obtained higher scores in both groups. The scores for the aforementioned diver’s certification were about 4 out of 5. For divers with the qualification of rescue diver or below, the scores were about 3 out of 5 for both groups. Those with the qualification of divemaster and instructor or above were found to be significantly associated with a higher awareness score of their own behaviour through ordinal regression. The odds of the instructor or above being considered to have a stronger awareness score to mind own behaviour was 7.260 (95% CI, 1.688 to 31.218) times that of the instructor and below, which is a statistically significant effect, Wald χ^2^ (1) = 7.095, *p* < 0.05. Moreover, the odds of the divemaster being considered to have a stronger awareness score to mind their own behaviours was 20.683 (95% CI, 1.966 to 217.606) times that of rescue diver or below, which is a statistically significant effect, Wald χ^2^ (1) = 6.365, *p* < 0.05.

Regarding the awareness score to mind others’ behaviours, the results from the ordinal regression showed that divemaster and instructor or above were linked to a significantly higher awareness scores to mind others’ behaviours. The odds of the instructor or above being considered to have a stronger awareness score to mind other’s behaviour was 7.208 (95% CI, 1.696 to 30.638) times that of the instructor below, which is a statistically significant effect, Wald χ^2^ (1) = 7.157, *p* < 0.05. Moreover, the odds of the divemaster being considered to have a stronger awareness score to mind others’ behaviours was 8.254 (95% CI, 1.323 to 51.497) times that of the rescue diver or below, which is a statistically significant effect, Wald χ^2^ (1) = 5.106, *p* < 0.05.

**Figure 9:**
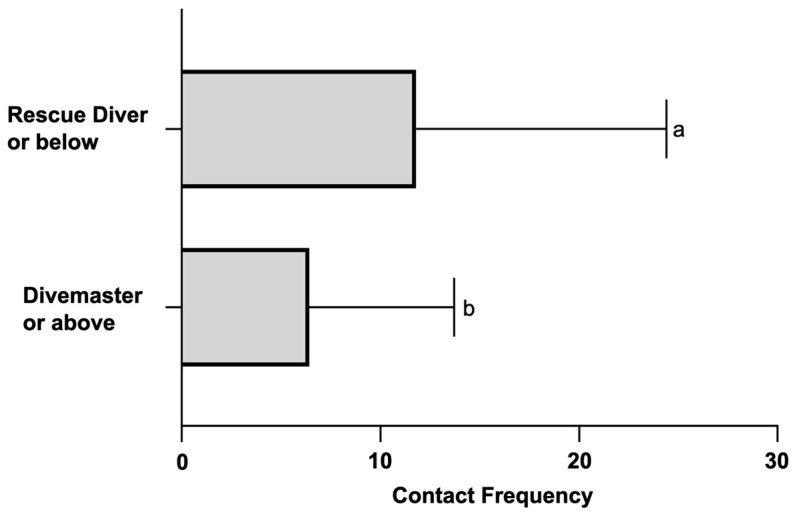
Contact frequency was grouped according to the diver’s certification level. ‘Rescue Diver or below’ includes the divers with the highest certification as open water diver, advanced open water diver, or rescue diver. ‘Divemaster or above’ only counts the divers with the highest certification as divemaster, instructor or other certifications above. Each error bar represents one standard deviation. A significant difference was found by the Mann–Whitney U test. Groups with a significant difference are indicated by different lower-case letters.

Divers with the qualification of rescue diver or below were pooled together for computing the mean and standard deviation of the contact frequency (Figure 9). Similar procedures were repeated to the group and yielded the summary values for divers with a divemaster or above qualification. Divers with a divemaster or above qualification had 6.4 ± 7.3 contact frequency, which was significantly lower than that of the group of rescue diver or below (11.8 ± 12.6 contact frequency) (Mann–Whitney U test, U = 535.5, *p* < 0.05).

## 4. Discussion

### 4.1. General Scuba Diving Behaviour Pattern of Recreational Divers

A total of 102 recreational divers, covering open water diver to instructor level or above, were recruited and observed for this study. Demographic data implied that most divers in Hong Kong are young to middle-aged with high education levels. This survey recorded a total of 1088 actual contact with marine biota, including 124 direct coral contact, inferring that at least one coral contact occurred in roughly every 10 contact with marine biota per dive. Coral damages, such as skeletal breakage, could happen with such contact [12], although we recorded no coral breakages during this study. One of the primary reasons why coral damage from divers in Hong Kong was assumed to have caused less damage is due to low coral coverage, and most of the marine substrates were comprised of sandy and muddy bottoms [12,24].

The most contacted body parts were hand and fin, accounting for 70% of the total contact. Our findings showed that more than 90% of the hand contact was intentional, and 73% of fin contact was unintentional. It demonstrated that recreational divers would hold on to corals or hard surfaces to stabilise themselves while diving, but they were unaware that their fins frequently make contact with the marine substrate [8,12,13]. It was also found that the use of a camera underwater drove intentional contact, as underwater photographers would attempt to hold onto corals or hard surfaces for stabilisation [8,12,58]. However, the camera was not a dominant cause of contact in this study. It may be due to the recruited divers’ prior knowledge of this diving impact survey. Therefore, it implied that the unnecessary contact related to the camera could be reduced if the divers were alerted before entering the water.

Our study on contact during different dive stages is consistent with the result of Chung et al., 2013 [12]. The contact number of the descent stage was nearly five times higher than the ascent stage, and there was also an inverse relationship between the contact number on the descent stage dive and the dive qualification. The underwater condition is also a potential factor, as one study recorded that the majority (96.7%) of the contact happened within the first 10 min of the dive [8]; the reason could be caused by poor visibility (altering predictions of the distance with the substrate) or strong water current (increasing tendency to hold onto substrates). Due to the frequent low visibility in Hong Kong waters, it is difficult to estimate the distance between the divers and the substrate during descent and the ignorance of the descending speed; inexperienced divers may directly land on the marine substrate [12]. It is recommended that the instructor/dive operator set up a descending rope for recreational divers, thus decreasing contact impact during descent.

### 4.2. Intention–Behaviour Gap of the Diving Behaviour

A direct inform approach was applied in this study to make sure the recruited divers were aware that they were being observed, unlike previous studies, which used an uninformed approach [12,58,59]. The 102 recreational divers were observed to have a total of 1088 contact with marine biota, with an average of 10.7 contacts per dive. The mean of perceived contact was 4.7 per dive from the 102 divers, which was almost half of the actual contact made and coincided with other studies on the existence of the intention–behaviour gap [49,50,51,52,53]. Some divers intended not to make contact with marine biota, but the actual behaviour was different from their expectations due to various technical or unknown issues. Some divers did notice that their actual behaviour was not what they perceived. When they responded to the follow-up questionnaire, they may still try to represent themselves in a morally acceptable manner because some may feel pressured or ashamed to describe their actual behaviour truthfully [49,60,61].

This study demonstrated a slightly different result than the previous study [12]. The mean of actual contact per dive in this study was lower than that of Chung et al., 2013 [12]. It is generally believed that the higher diver certification level should come with better diving skills, hence less contact with marine biota. Given that this study covered more divers than the other studies, it provided a more accurate picture to understand how the diver profiles and diving experiences influence the contact number with marine biota, as similar studies were conducted on the small sample sizes of divers and dominated by beginner-level divers [12,59].

As reported in previous studies, the diver group with rescue diver level or below tended to cause more contact than the dive leadership group with the divemaster level or above [8,12,58]. Our study interestingly showed that even the rescue divers made a similar contact rate as beginner divers. The divemaster level or above group had a significantly higher mean score in environmental awareness, as experienced divers can pay more attention to the diving behaviour of themself and others. The divemaster course requires all candidates to have an internship assisting instructors during the dive-training procedure, and the course content and examination framework covered the basic knowledge of marine ecology and conservation [62].

Our findings suggest that the concepts related to marine conservation and coral ecology are recommended to be added to the compulsory course outline and examination of the Open Water Diver course to further enhance awareness of beginner-level divers. The environmentally responsible speciality courses and diving programmes from different scuba diving training agencies are also a possible way for scuba divers to have more understanding of marine conservation and thus address diving impacts, such as the Coral Reef Conservation course from PADI Project AWARE, Underwater Ecologist (Coral Reef) Specialty from The National Association of Underwater Instructors (NAUI), Coral Identification Specialty from Scuba Schools International (SSI) and Coral Conservation Specialty course from Scuba Diving International (SDI) [58,62].

### 4.3. Implication of Citizen Science

The citizen science project is an effective method for mass data collection within a short period, providing sufficient time for data sorting and analysis [63,64,65]. Although citizen science may give rise to a survey bias because of insufficient training and varied ability, this effect was minimised with careful selection and repeated pre-survey training as outlined above [65,66].

It is a win-win situation when utilising citizen science in a conservation approach [27,49,63,64,67]. It enabled WWF-HK to shorten the data collection time, while participating citizen scientists gained more knowledge on ocean ecology and diving techniques [65,68]. Paying particular attention to the damaging behaviours of recreational divers by these experienced instructors also raised their awareness of investing more effort into teaching buoyancy and finning skills. With that being the case, not only can resource-limiting NGOs collect a large amount of data in a short time, but instructors could also gain experience in mobilising ocean conservation events, which can encourage them to participate in similar conservation events in the future.

This research also used citizen science as an essential strategy for awareness raising [69]. Citizen scientists could experience and visualise the negative impacts brought by divers to the ocean, which possibly stimulated them to reflect on the relationship between humans and nature, thus leading to more conservation behaviours [68,69]. More importantly, as a large proportion of the citizen scientists were instructors or even course directors from different dive-training agencies, the concept of “train the trainer” was applied to the training [70]. Well-trained citizen scientists could pass what they had learnt to their students in the future, expanding the influences of this project from specific scientific research to general conservation awareness education. The success of this research proved that citizen science could be applicable to different scientific research that has large data demand and limited resources, but sufficient guidelines and training must be given to the participants [65,66]. Given that the citizen science approach benefits both researchers and participants, it plays an important role in promoting scientific research to the general public by including public participation in the data collection process [49,64,66,69].

### 4.4. Education and Policy Implication

Scuba diving usually takes place at marine ecological hotspots and marine protected areas. To address the visiting stress brought by recreational scuba diving, WWF-Hong Kong launched the “ECF- Dive for Ocean” project in 2021 to promote ocean-friendly scuba diving. The programme was designed to have critical community engagement elements. A pioneering attempt locally is the production of a set of dive-training cue cards with conservation messages, which was co-developed together with 80 experienced instructors and course directors from the four main scuba diving agencies worldwide (PADI, NAUI, SSI and SDI). The cue cards also contained a coral distribution map of the most popular dive-training site, Sharp Island West, in Hong Kong, ocean-friendly training instructions and information on the relevant corals. They are helpful training tools for instructors, assisting them to be mindful when selecting suitable training sites, equipping their dive trainees with basic coral knowledge, and ensuring they adhere to a code of conduct to keep a distance from marine biota. A communication campaign, “Mind Your Fins”, was also conducted. The project, which aimed to boost further the community’s awareness of minimising physical contact with marine biota and substrate, featured videos with influencers or Key Opinion Leaders (KOL) and a viral social media campaign targeting the scuba diving community. Over 2000 dive trainees received dive training incorporating the aforementioned code of conduct during the 15-month project period. The launch of this pilot project illustrated the significance with which conservation can be integrated into scuba diving training with strong stakeholder support, especially from the scuba diving industry leaders.

Given the intention–behaviour gap of the diving activity, disturbance to the marine biota by recreational activities cannot be fully addressed solely by public education. Recreational activity stress, in addition to other human disturbances and disastrous natural events, may drastically deteriorate the health of ecological hotspots and marine protected areas in the long term if not managed properly. Well-managed marine protected areas (MPAs) are considered effective policy instruments to address various pressures on marine biodiversity, including stress from recreational water sports activities [71]. To ensure MPAs are effectively managed, it is recommended that conservation objectives should be clearly defined according to the site-specific marine ecological value and threats. Progress and management effectiveness should be evaluated against long-term monitoring protocol [71,72]. Marine carrying capacity should also be addressed through site management, visiting regulations, and carrying capacity enhancement methods to drive sustainable diving tourism and resilient ecosystems to safeguard the coral communities in Hong Kong.

## 5. Conclusions

This study updated the scuba diving behavioural information in Hong Kong using a citizen science approach and the differences between the actual behaviour from the direct observation survey and the perceived behaviour from a self-reported questionnaire in the context of recreational scuba diving. Considering the budget shortage and cost-effectiveness, citizen science is an effective way to collect more data samples and shorten the data collection process. Citizen science-based ecological monitoring has been commonly applied to various long-term biodiversity monitoring [49,63,64,65,66,67,68,69]. This study demonstrated a case study for scientists and NGOs on utilising the citizen science approach to conduct a good quality study and achieve the purpose of public awareness raising.

Scuba divers have generally shown a high environmental awareness from the questionnaire result, while the overall mean score was 3 out of 5. However, it may not directly reflect their actual behaviour. Regarding the transition from perception to actual, an intention–behaviour gap was found between perceived and actual diving behaviour, showing that recreational divers may not fully know to control their descending speed and their unintentional contact while diving. Additionally, diving qualifications and experience significantly govern the contact rates.

With the support of the diving industry, WWF-Hong Kong has developed a series of voluntary conservation tools for instructors to empower instructors in promoting ocean-friendly diving behaviour, and to fill in the information gap in current dive-training courses on ocean conservation. Additionally, education could pose a significant difference in the contact rate as pre-dive briefings and warnings are effective in alerting divers to monitor their behaviours [9,30,43]. Consequently, the tools are aimed to remind the divers to reduce the number of contacts underwater. To address the impacts of marine-based recreational activities holistically and prevent the loss of coral areas, we recommend that the government of HKSAR should set up well-managed Marine Protected Areas by providing adaptive management plans for popular recreational diving areas and facilitating sustainable nature-based tourism with stakeholder engagement of the scuba diving industry in the major coral areas.

## Figures and Tables

**Table 1:** Divers’ profiles were summarised below.

	Count
Gender	
Male	53
Female	49
Age Group	
20–34	58
35–49	35
50 or above	9
Academic Level	
Secondary	20
Post-secondary	24
Tertiary	41
Post-graduate	17
Monthly Income	
Unknown	19
Below HKD $9999	3
HKD $10,000–19,999	11
HKD $20,000–39,999	47
HKD $40,000–59,999	18
HKD $60,000–79,999	3
HKD $80,000 or above	1
Divers’ Qualification	
Open Water Diver	17
Advanced Open Water Diver	46
Rescue Diver	18
Divemaster	7
Instructor or above	14

## Data Availability

Not applicable.

## Appendix A. Follow-Up Questionnaire

**Part (1) Personal Information**									
**1. Gender**		Male	Female						
**2. Age Group**	19 or below	20–34	35–49	50 or above					
**3. Education Level**	Primary	Secondary	Undergraduate	Postgraduate	Degree				
	Master or above								
**4. Monthly Income**	Below $9999	$10,000–19,999	$20,000–39,999	$40,000–59,999	$60,000–79,999				
	$80,000 or above	No income (retired or student)		Not feasible to disclose					
**Part (2) Dive Experience**									
**1. Which diving certification agency did you obtain your qualification from?**	PADI	SSI	SDI	NAUI					
**2. Which of the following is your highest diver qualification?**	Open Water Diver	Advanced Open Water Diver		Rescue Diver	Divemaster				
	Instructor or above								
**3. Dive experience (Number of dive log)**	1–25	26–50		51–100	101–200				
	201–500	500 or above							
**4. How much do you spend on your dive equipment annually?**	$1999 or below	$2000–6999		$7000–11,999	$12,000–16,999				
	$17,000–21,999	$22,000–26,999		$27,000–31,999	$32,000–36,999				
	$37,000 or above								
**5. Before the pandemic, how many times do you dive per year on average in Hong Kong?**	0–4 times	5–10 times		11–20 times	21–30 times				
	31–40 times	41–50 times		more than 50 times					
**6. How much do you spend per diving trip in Hong Kong?**	$299 or below	$300–399		$400–499	$500–599				
	$600–699	$700–799		$800 or above					
**7. How many overseas diving trip have you had in total?**	Never	1–5 times		6–10 times	11–20 times				
	21–30 times	31–40 times		40 times or above					
**8. Before the pandemic, how many overseas diving trip do you go on average annually?**	Never	1–2 times		3–4 times	5–6 times				
	6 times or above								
**9. How much do you spend per overseas diving trip on average?**	Below $9999	$10,000–19,999		$20,000–29,999	$30,000–39,999				
	$40,000–59,999	$60,000–69,999		$70,000 or above					
**Part (3) Diving Behaviour**									
**Please click the numbers that indicate to which extend will you do the following action (−2 = to a very small degree, −1 = to a small degree, 0 = neutral, 1 = to a large degree, 2 = to a very large degree)**									
**1. How often do you dive with awareness of how your diving behaviour (e.g., swinging your fins) affects nearby marine life (like coral colonies)?**					−2	−1	0	1	2
**2. How much do you pay attention to the impact of other people’s diving behaviour (such as swinging fins) on nearby marine ecology (such as coral colonies)?**					−2	−1	0	1	2
**3. To what extent do you think scuba diving activities can harm corals or other marine life?**					−2	−1	0	1	2
**4. As a diver, to what extent do you feel it is your responsibility to protect the environment on your dive site?**					−2	−1	0	1	2
**5. Do you think you contacted marine substrate when you were diving just now?**		Yes		No					
**6. How many times, if any, you thought you have contacted the sea floor during your dive just now? (Please fill in the number)**		_____________________________________							
**7. What part of your body do you think is mainly in contact with the marine biota or substrate?**	Hand	Knee		Fin	Air tank				
	Second regulator	Diving Camera		Other					
**8. Which type of marine substrate do you think you have contacted**	Mud	Sand		Rock	Coral				
**9. Do you think you have directly/indirectly caused coral damage in the dive just now?**	Yes			No					
**10. Which of the following is/are the cause of contact with coral or other marine life for you?**	The current is too strong	Improper buoyancy control		When taking photos	Got influenced by other divers				
	Low visibility	Other							
